# A Grain-Scale Study of Mojave Mars Simulant (MMS-1)

**DOI:** 10.3390/s21144730

**Published:** 2021-07-10

**Authors:** Sathwik S. Kasyap, Kostas Senetakis

**Affiliations:** Department of Architecture and Civil Engineering, City University of Hong Kong, Kowloon, Hong Kong, China; ssarvadev2-c@my.cityu.edu.hk

**Keywords:** mars simulant, vesicular texture, friction, grain-scale mechanics, space exploration, micro-scale experimentation

## Abstract

Space exploration has attracted significant interest by government agencies and the scientific community in recent years in an attempt to explore possible scenarios of settling of facilities on the Moon and Mars surface. One of the important components in space exploration is related with the understanding of the geophysical and geotechnical characteristics of the surfaces of planets and their natural satellites and because of the limitation of available extra-terrestrial samples, many times researchers develop simulants, which mimic the properties and characteristics of the original materials. In the present study, characterization at the grain-scale was performed on the Mojave Mars Simulant (MMS-1) with emphasis on the frictional behavior of small size samples which follow the particle-to-particle configuration. Additional characterization was performed by means of surface composition and morphology analysis and the crushing behavior of individual grains. The results from the study present for the first time the micromechanical tribological response of Mars simulant, and attempts were also made to compare the behavior of this simulant with previously published results on other types of Earth and extra-terrestrial materials. Despite some similarities between Mars and Moon simulants, the unique characteristics of the MMS-1 samples resulted in significant differences and particularly in severe damage of the grain surfaces, which was also linked to the dilation behavior at the grain-scale.

## 1. Introduction

The China National Space Administration (CNSA), the National Aeronautics and Space Administration of USA (NASA) and the European Space Agency (ESA), among other government and regional agencies, have initiated many space exploration research programs in an attempt to investigate the geological and environmental conditions on the Moon, the surface of the Mars and other planets so that to provide feasibility studies on possible future colonization. This has resulted in enormous progress in recent years in the development of technologies related with space exploration and the scientific study of planets, the mapping of their surfaces and the potential form of life out of Earth [1,2,3,4,5,6,7,8,9,10,11], in which astrobiology research has also provided substantial contributions [12]. Understanding of the surface conditions and the mechanical properties of planets and their natural satellites is critical to be obtained as it will affect the potential design of facilities and provide an understanding of roving vehicle–ground interaction [13,14,15]. However, it has been challenging to study these problems as the availability of original extra-terrestrial materials is very limited and for this reason researchers have developed simulants which mimic the real surface properties of extra-terrestrial geological materials [16,17,18,19]. With respect to Mars regolith, a number of simulants have been presented in the literature, for example, the JSC Mars-1 by Allen et al. [17], the MMS by Peters et al. [20], the SSC-1 by Scott and Saaj [21], the JMSS-1 by Zeng et al. [22], the MGS-1 by Cannon et al. [23] and the PSI HX by Wang et al. [24], and different characterizations have been carried to compare the simulants with the original regolith. Some of the major mechanical properties of simulants such as compressibility, friction (strength), and seismic velocities have been explored to provide a comprehensive characterization on their geotechnical and geophysical properties [25]. One of the recent Mars simulants (ESA^2^C) developed by Smith et al. [26] was characterized in terms of chemical, grain shape and strength parameters, while most of the simulants were only chemically characterized to be compared with the original Mars regolith. Mechanical characteristics, such as friction, are essential for the regoliths, as they affect the performance of rovers in the vehicle test beds and on Mars [21]. The grain-scale analysis of Mars simulants, including shape characteristics and micromorphology, is also essential to study transport processes on Mars’ surface [27].

Despite these efforts, only a limited amount of works has focused on the mechanical characterization of Mars simulants [25,26,28], and these works have been directed, predominantly, on their macroscopic (bulk) behavior. However, a complete characterization of the constitutive behavior of geological materials necessitates their examination at multi-scales, including the interactions between grains (i.e., micromechanical response) and the influence of these interactions on the bulk behavior of “granular” systems. For example, previous studies on granular materials have shown a strong dependency of their mechanical response as bulk systems, on the interparticle friction [29,30,31,32,33] and the properties/characteristics of individual grains such as roughness, shape and strength/mode of failure, which influence the energy dissipation mechanisms and the resistance against shearing-induced strains [34,35,36,37,38,39,40,41,42]. Multi-scale insights of granular systems can provide a framework to understand the load transfer mechanisms and the flow behavior of particulate materials at a fundamental level [36,37,38,43,44,45,46,47,48,49,50,51,52,53,54,55,56,57,58], which are strongly linked to the properties at the grain-scale. Thus, based on what the state-of-the-art literature suggests, quantification of the grain-scale interface behavior and morphological/mechanical characteristics of individual particles of extra-terrestrial materials, would be invaluable to produce predictive tools which will allow a precise examination of rover wheel-soil interaction, structure-soil interaction as well as a fundamental understanding of transport processes on the surface of the Mars and the Moon. This can have implications in both design of engineering systems and geophysical studies in relation to space exploration. 

Multi-scale insights on the behavior of lunar regolith simulant (DNA-1A) were recently obtained in the studies by [59,60]. These works showed a strong dependency of the grain-scale characteristics and mechanical behavior, such as interface friction, on the bulk response of the simulant and they also provided comparisons with a benchmark quartz-based sand. In the present work, a pilot study on the micromechanical behavior of MMS-1 Mars regolith simulant is presented based on grain-scale experiments, with major objectives to (i) Characterize the material in terms of morphological characteristics and composition; (ii) Quantify the mechanical behavior of the Mars regolith simulant in terms of particle strength and mode of failure as well as by means of frictional (or tribological) behavior. The results from the present study are compared with previous works on Mars and Lunar regolith simulants and attempts were made to provide some multi-scale insights, contributing to the state-of-the-art knowledge on the properties and behavior of extraterrestrial materials with potential applications in space exploration. Outcomes from the present work can be directly used in discrete-based simulations of Mars surface and the study of rover wheel-soil interaction contributing in this way to the geophysical and geotechnical research with respect to space exploration.

## 2. Material, Equipment and Methodology

### 2.1. Material Description

**Figure 1:**
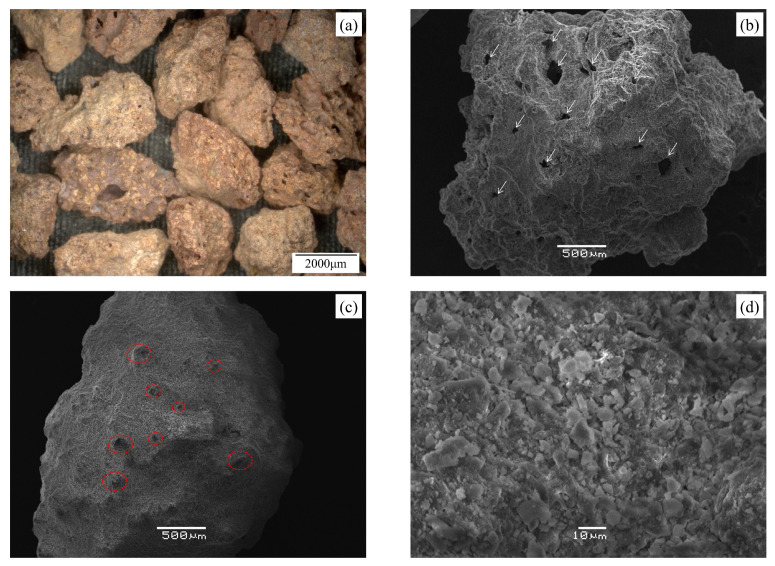
MMS-1 Mars regolith simulant: (**a**) Microscopic image of representative particles. SEM images of representative MMS-1 grains highlighting (**b**) pores, (**c**) protrusions and (**d**) natural surface coating.

The commercially procured Mojave Mars Simulant (MMS-1) was used for the micromechanical tests, which were directed on grain-to-grain frictional experiments and grain crushing tests. This simulant was produced from high-quality, iron-rich basaltic rocks and a 2.36–5.00 mm fraction was used in this study (Figure 1a). The average values of the smallest circumscribing cuboid dimensions (after [61]) were measured to be equal to 2.95, 2.31, and 1.95 mm in three perpendicular directions. The MMS-1 grains are reddish to brown in color, highly irregular in shape, and their structure is formed by weakly bonded fine to coarse silt-sized particles and a coating of dust. Grains with average sphericity and roundness values (after [62]) greater than 0.6 were hand-picked for the micromechanical tests. The shape of a given grain (using images) from each of the three orthogonal directions (two in the horizontal plane and one top view) are matched with the reference shapes provided in the chart by [62]. The corresponding sphericity and roundness for each selected shape in the chart are recorded, and the arithmetic mean of the values from the three directions are considered as the shape descriptors for a given grain. Researchers also used more advanced imaging techniques such as X-ray computer tomography to accurately quantify the shape descriptors (e.g., [63]). Even though the overall grain shape is critical in the behavior of geological materials, the current micromechanical testing on a single grain pair (i.e., two grains in peak-to-peak contact) and the microstructural features near the contact zone are highly influential, which are particularly necessary to be assessed for damage analysis (Section 3.2.4).

The MMS-1 grains are similar to DNA-1A lunar regolith simulant in terms of grain shape. Sandeep et al. [59] described the DNA-1A grains as subangular to angular in shape based on Powers [64] chart. Based on microstructure-textural characterization from scanning electron microscope (SEM) images (FEI/Philips XL30 Esem-FEG, Hillsboro, OR, USA), two different classes of MMS-1 grains were distinguished. The first class of grains is highly vesicular (Figure 1b) with visible pores having an area as large as 0.045 mm^2^ (bounding rectangles of approximately 0.25 × 0.18 mm in size). The second class of grains is characterized by small amounts of identifiable pores with protrusions of coarse silt-sized grains leading to irregular surface peaks (Figure 1c). SEM images at higher magnification showed that the surface coating is majorly composed of very-fine flaky particles (<10 μm) (Figure 1d).

Using white light interferometry analysis (Veeco NT9300 optical surface profiler, after Sandeep et al. [65]), a representative set of 15 surface roughness (S_q_) measurements was performed on five different grains and the average value (which expresses an RMS roughness) was measured to be equal to 2087 nm with a standard deviation of ±985 nm for a scanned area of around 400 μm^2^. As a comparison with other materials of similar textural features (i.e., porous structure), the S_q_ value of DNA-1A (lunar regolith simulant) and pumice were reported to be equal to 1476 ± 379 nm (after [59]) and 2390 ± 620 nm (after [66]), respectively. These roughness values are much higher compared with other geological materials such as Ottawa sand particles (204 ± 42 nm, after [59]), Leighton Buzzard sand (LBS) quartz particles (223 ± 61 nm, after [67]) or crushed limestone particles (670 ± 221 nm, after [67]).

Energy-dispersive X-ray spectroscopy (EDS) analysis was also performed (in conjunction with the SEM) to identify the percentage weights of a given mineral, and the values are compared with other Mars simulants reported in the literature [20,68,69]. In the present study, five different MMS-1 grains with different grain-scale morphologies (as shown in Figure 1) were analyzed, and the corresponding element weight percentages are shown in Table 1. The presence of pores (Figure 1b) or protrusions (Figure 1c) on the MMS-1 sample did not reflect major deviations in the percentage element weights. Similar to other Mars simulants reported in the literature, Si and O were the dominant elements in the present sample. However, significantly higher percentages of Fe (and corresponding oxides) were observed in the present sample compared to MMS and JSC Mars-1 as reported by Peters et al. [20] and MMS-1 samples by Caporale et al. [69], which might lead to a higher coefficient of static friction (after Noh and Jang [70]). However, it is noted that the specific methodology adopted for the quantification of elements (and their oxides) in each of these studies is different, leading to differences in the measured quantities. In their corresponding mineralogical examinations, Peters et al. [20] did not find any clay minerals in the MMS samples they examined, while Morris et al. [68] found 1% weight of clay minerals. Caporale et al. [69] reported the presence of smectite minerals (possibly Na-saturated montmorillonite) based on XRD peaks of <2 μm MMS-1 sample.

### 2.2. Testing Apparatus and Methodology

A micromechanical testing apparatus recently developed by Kasyap et al. [71] was used in this study to investigate the tribological behavior of MMS-1 grain contacts in terms of normal and tangential responses. The apparatus consists of two loading systems in the vertical and horizontal directions which implement the normal and tangential loadings to the grain contacts, respectively. Each loading system is composed of a linear actuator (60 mm travel range, 0.198 µm micro-step size), a load cell (±100 N capacity, ±0.06 N precision) and a non-contact displacement transducer (±3 mm range, ±0.04 μm precision), which are connected with stiff mechanical components to reduce the compliance of the apparatus in both the horizontal and vertical directions. Note that even though there is a tolerance of the precision of the sensors (load cells and displacement sensors) in the measurement of the dynamic contact friction (i.e., interparticle friction during steady-state sliding), accurate measurements of forces at the level of millinewton and displacements at the level of micrometer are necessary to resolve for contact stiffness in the analysis of the grain-to-grain contact response for small size particles [72,73,74]. Thus, the use of high precision sensors and high-quality amplifying and data logging systems along with low-pass filters are necessary to smoothen the data and derive grain-scale parameters from the experiments. This is particularly important in micromechanical modeling, as the contact stiffness is a core input parameter in discrete-based numerical simulations, however, less attention has been given in the experimental analysis of both normal and tangential grain-scale stiffness of extra-terrestrial materials.

**Figure 2:**
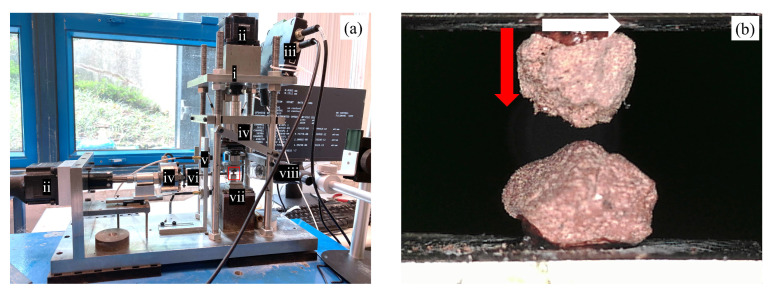
Experimental setup: (**a**) Micromechanical loading apparatus with its different components marked (i) rigid frame (ii) linear actuators (iii) motor controllers (iv) load cells (v) non-contact displacement transducers (vi) shear loading arm (vii) rigid base (viii) digital micro-camera. (**b**) Close view of particle arrangement for micromechanical testing (red arrow indicates normal loading and white arrow indicates tangential loading).

The apparatus is controlled using a set of commands instructed through a custom-built code connected to various mechanical parts of the apparatus, which continues to operate based on the feedback from the sensors. The individual components of the apparatus are described in Figure 2a, and Figure 2b shows a closer view of a representative MMS-1 specimen arranged for grain-to-grain interface testing (the image corresponds to a specimen just before the grains are set in contact). The top grain is glued to a mount fixed to the vertical loading system, which is also rigidly connected to the horizontal loading system through a combination of linear bearings for swift motions. The top grain can move towards and away from the fixed lower grain in the vertical direction for the imposition of the loading and unloading phases, respectively. Once the required (or nominal) normal load is reached at the contact of the grains, the top grain is then sheared against the bottom grain under a given constant normal load. The directions of normal and tangential loads are indicated in Figure 2b.

In the present study, the normal and tangential contact response of MMS-1 grains was examined at a nominal normal load (F_N_) of 2 N (note that the actual normal load slightly deviated from the nominal value during the experiments because of the highly irregular morphologies of the grains). After the required F_N_ was reached, shearing was applied to the grain pairs for a sliding displacement of around 100 μm. Both the normal and tangential loads were applied in a displacement-controlled mode, and the constant normal load during shearing was maintained in a force-controlled mode to monitor the vertical deformation during shearing. Both monotonic and repeated monotonic loading tests were conducted with these specifications. In monotonic loading tests, each specimen was subjected to the required normal force and, consecutively, shearing was applied to the grain contact. In repeated monotonic loading, each specimen was subjected to three cycles of normal loading and shearing, i.e., after the application of the nominal F_N_, the specimen was subjected to shearing for the required sliding path and consecutively, the pair of grains was set to their reference position and a second (and a third) cycle of normal loading and shearing was applied so that to maintain the same contact region among all the cycles. Based on the experimental protocol for the repeated loading tests, the influence of loading history could be examined, similar to previous studies on various types of geological materials [75,76,77]. As different MMS-1 specimens showed highly varied normal and tangential contact responses, monotonic shearing tests were performed on 12 specimens and three cycles of repeated loading tests were conducted on a representative set of two specimens. Table 2 shows the testing details and key outputs from the experiments.

**Figure 3:**
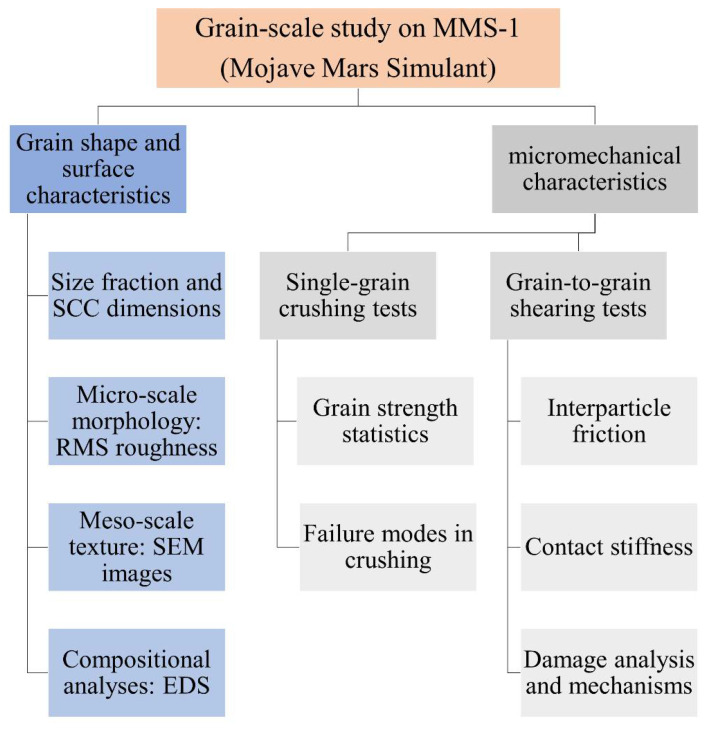
Illustration of the workflow in the present grain-scale study on MMS-1.

A representative set of single grain crushing tests were also conducted on MMS-1 particles to provide additional characterization of the material in terms of particle strength, and the crushing strength characteristics of the MMS-1 simulant are compared with that of other types of geological materials in the subsequent section. The load-displacement variations in the normal and tangential directions (F_N_-δ_N_ and F_T_-δ_T_) are particularly highlighted in this study, along with the damage analysis of the MMS-1 grain surfaces, and the variation of the normal displacements (δ_N,shear_, compression or dilation) during shearing for different pairs of grains. The flowchart of Figure 3 provides a summary of the whole process of material characterization and the grain-scale experiments with their respective primary outputs.

## 3. Results and Discussion

### 3.1. Single Grain Crushing Strength

**Figure 4:**
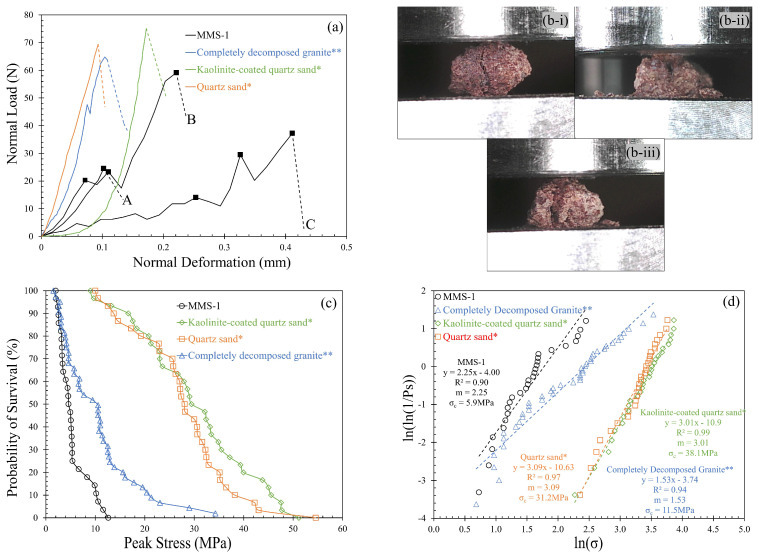
(**a**) Variation of normal load against normal deformation; (**b**) Modes of crushing observed for MMS-1: (**b-i**)-Splitting, (**b-ii**)-Significant fragmentation, (**b-iii**)-Mixed; (**c**) Variation of survival probability with peak stress; (**d**) Determination of Weibull modulus and characteristic strength. (* Kasyap and Senetakis [81]; ** Wang and Coop [80]).

Single grain crushing strength has been used as a micromechanical fundamental property for various geological materials [78,79,80,81]. A representative set of 15 single grain crushing tests was performed on MMS-1 grains (similar size fraction of 2.36–5.00 mm) using a modified CBR apparatus (after [80,81,82]). The single grain crushing characteristics of MMS-1 were compared with three different geological materials from the literature; (i) completely decomposed granite of 1.18–2.36 mm fraction tested by Wang and Coop [80] (ii-iii) natural and kaolinite-coated quartz sand grains of 2.36–5.00 mm fraction tested by Kasyap and Senetakis [81]. A representative set of normal load-deformation curves of these grains are shown in Figure 4a. The normal load-deformation curves for quartz sand and kaolinite-coated quartz sand were scaled down four times to match the normal load range of other materials described in Figure 4a.

The MMS-1 samples tested in this study crushed in a wide range of normal loads and deformations. The maximum and minimum values of peak load are 14.8 N and 84.6 N, and the respective limiting deformations at failure are 0.102 mm and 0.411 mm, respectively. Within this wide range of crushing loads and deformations, three classes of load-deformation curves (denoted as A, B, and C in Figure 4a) were identified and a mode of crushing is associated with each class, as shown in Figure 4b. The grains that were crushed at very low normal loads of less than 30 N (class A in Figure 4a) tended to show a distinctive splitting behavior through the grain body (Figure 4b-i) without any noticeable fragmentation during the loading process. The other class of grains showed comparatively higher normal loads but with significant fragmentation throughout the loading process (Figure 4b-ii), leading to extended deformations prior to failure. The grains with significant vesicles or surface pores showed this trend of failure. The third class of grains showed comparatively higher crushing loads and stiffer load-deformation response while only minor fragmentations were observed. The failure mode for this class of grains was mixed with fragmentation and splitting (Figure 4b-iii). The initial fragmentation led to slight drops in the normal load, and consequently, a stable contact was established between the MMS-1 grain and the loading platens, leading to higher crushing loads. A similar classification of normal load-displacement curves from single grain crushing tests on pumice was reported by Orense et al. [83].

Based on the load-deformation curves, the comparison of MMS-1 grains with other geological materials signifies the influence of vesicles in the MMS-1 grains. The completely decomposed grains also show some indications of fragmentation in the load-deformation curves, but the three materials compared in Figure 4a show stiffer load-deformation response compared to MMS-1 grains. Further comparison of MMS-1 grains with these geological materials can be made based on statistical parameters used for the evaluation of the crushing characteristics, such as survival probability, Weibull modulus and characteristic strength. Based on the crushing load and smallest circumscribing cuboid dimensions (after [61]), the crushing strength of the grain can be calculated as the maximum tensile stress based on Equation (1) (after [79]).

(1) $$\sigma_{p}=\frac{0.9*F_{c}}{d^{2}}$$

In Equation (1), *F_c_* is the crushing load, and d is the geometric mean of the intermediate and smallest diameters of the grain. Within the population of the grains tested, the probability of a grain to survive (*P_S_*) a given peak stress is calculated based on Weibull statistics [84], according to Equation (2). The survival probability versus peak stress variation for MMS-1 grains is plotted in Figure 4c, and the corresponding variation for other geological materials is also compared in this figure. The range of crushing strength values for MMS-1 grains is from 2 MPa to 12 MPa; this range of values is comparable with that of CDG grains for stresses lowers than 5 MPa, however, the values deviated significantly in the regime of higher stresses. Kasyap and Senetakis [81] concluded that the natural and kaolinite-coated quartz sand grains showed a similar trend of survival probability despite the surface layer being coated with soft kaolinite. The major difference between coated and uncoated grains was observed only in the normal load-deformation behavior in terms of the initial regime of behavior (with extended displacements to be observed for coated grains to reach peak stress). Even though the MMS-1 grains have micro-particles adsorbed on their surfaces, the weak and highly vesicular structure of MMS-1 grains resulted in a lower survival probability.

(2) $$P_{S}=\exp\left(-{\left[\frac{\sigma_{p}}{\sigma_{c}}\right]}^{m}\;\right)$$

The single grain crushing characteristics of MMS-1 are further explored in terms of characteristic strength, *σ_c_* (37% of the population possess higher or equal strength than this value) and Weibull modulus, *m* (signifying variability of the strength of the grains within the population; higher values indicate lower variability). Applying the natural logarithmic function on both sides of Equation (2) leads to a linear function, where the slope represents the Weibull modulus, and the characteristic strength can be calculated from the intercept Equation (3).

(3) $$\ln\left(\ln\left(\frac{1}{P_{S}}\right)\right)=m\ln\left(\sigma_{p}\right)-m\ln\left(\sigma_{c}\right)$$

Figure 4d shows the linear fitting, R^2^, Weibull modulus and characteristic strength for the four materials. The characteristic strength of MMS-1 grains was 5.9 MPa, which value is almost two times smaller than that of CDG and five times smaller than that of the quartz sand variants (i.e., clay-coated and natural particles). The Weibull modulus of MMS-1 grains (*m* = 2.25) is lower than that of the quartz grains (*m* = 3.1) but higher than that of CDG (*m* = 1.53). These Weibull modulus values signify that the quartz grains have highly uniform strength within the population while the CDG grains have the least uniformity. MMS-1 grains showed an intermediate value, and it is to be noted that the Weibull modulus and characteristic strength values are slightly affected by the size of the population tested.

### 3.2. Normal and Tangential Contact Response of MMS-1 Grains

#### 3.2.1. Normal Contact Response: Monotonic Loading

**Figure 5:**
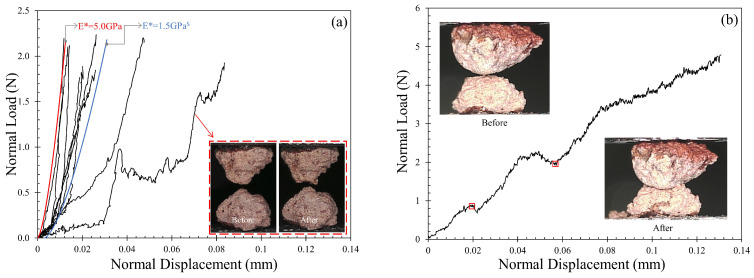
Normal contact response of MMS-1 grains (**a**) at maximum load of 2 N with an indication of contact modulus range and (**b**) at maximum load of 5 N (^$^ initial shift in normal displacement applied).

Normal loading for the MMS-1 specimens was applied at a rate of 0.2 mm/h until the required normal load was achieved (equal to 2 N for most tests) and representative plots of normal load–displacement curves are given in Figure 5. The results indicated that the normal contact response of MMS-1 specimens was highly dependent on the structure of the grains. As described in Section 2.1, some MMS-1 grains have a highly vesicular structure which collapses upon loading. The normal contact response of this category of grains was identified with extended compression of the order of 45 μm to 90 μm (Figure 5a) to reach a given normal load of 2 N, and some specimens underwent severe damage during the normal load application (inset of Figure 5a); these specimens were not further considered for tangential loading tests in this study. However, the majority of the specimens showed only slight irregular trends in the normal load-displacement curves due to the plastic deformation (or breakage) of the asperities and collapse of micro-vesicles within the contact zone of the grains during the loading process.

Researchers observed similar irregularities in the normal contact response of geological materials such as pumice [66] and silt-coated quartz sand particles [85]. Combining these observations with previous studies on various types of geological materials [66,77,85,86,87], these high undulations in the normal contact response curves can be ascribed, predominantly, to the porous and highly irregular morphology of the grains; additionally, the coarse-grained texture of the MMS-1 grains (assembled by smaller particles) could have also contributed to this behavior, which would be less possible for fine-grained textures (e.g., uniform coating of clay-type microparticles).

In Figure 5a, analytical curves from Hertz normal contact model (after Hertz [88]) are also plotted to indicate the range of contact modulus of MMS-1 grains (benchmark model curves with contact modulus, E^*^, of 1.5 and 5.0 N are illustrated in this figure). It is noted that Hertz model may not be the optimum model to be used for highly irregular and vesicular-in-texture grains such as MMS-1, but a qualitative understanding can be accomplished on the range of contact modulus for MMS-1 grains. Most of the curves for the MMS-1 specimens were located within E* values of 1.5 GPa and 5.0 GPa, which values are generally lower than that of DNA-1A lunar regolith simulant (average reported value of around 7 GPa) tested by Sandeep et al. [54] and kaolinite-coated quartz sand particles (average reported value of 5.9 GPa) tested by Kasyap and Senetakis [77].

A representative set of two specimens was tested at normal loads greater than 2 N to observe the contact response at higher normal loads. The gravity in Mars (3.72 m/s^2^) is lower than Earth’s gravity (9.81 m/s^2^), and hence the packing of the grains on Mars is expected to be relatively loose [24], so that testing under lower normal loads has more practical applications than testing at higher normal loads. However, to understand the behavior of MMS-1 grains at higher normal loads, an attempt was made to increase the normal load beyond 2 N. The characteristic crushing strength of MMS-1 grains was found to be 5.9 MPa (Section 3.1), which corresponds to a 40 N normal load on a single grain. For grain-to-grain contact tests, because of the developed smaller contact area, the stresses are expected to be higher and hence, significant crushing of the grains was observed at normal loads greater than 5 N (Figure 5b) with total deformations of about 130 μm. This crushing load is only nominal as it depends on the local radius and hence the contact radius of the grain pair. The fragments generated during the loading process were further crushed between the top and bottom grains as the normal load increased.

#### 3.2.2. Tangential Contact Response: Monotonic Loading

**Figure 6:**
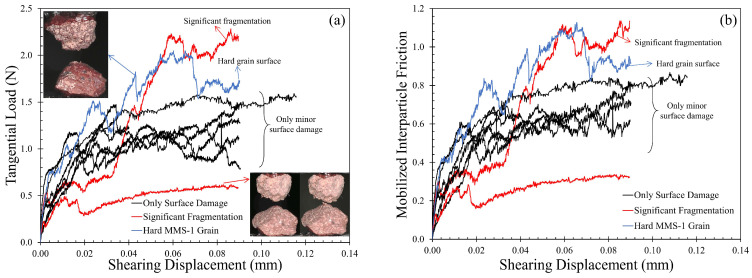
Tangential contact response of MMS-1 grains, (**a**) tangential load and (**b**) mobilized interparticle friction against shearing displacement.

After the required normal load was attained, tangential loading was applied on the grain contacts at a rate of 0.1 mm/h under a constant (nominal) normal load of 2 N, and representative plots from the experiments are shown in Figure 6. Similar to the normal contact response, the behavior of the specimens in the tangential (or shearing) direction was highly irregular (Figure 6a), with significant fluctuations in the tangential load due to asperity breakage and collapsing of the vesicles.

During the sliding process, a few MMS-1 specimens showed a sudden drop in the tangential load due to significant fragmentation. Two such cases are highlighted in Figure 6a, and a representative image of the grain pair in shearing is also shown in the same figure. The post-breakage shearing behavior is unrestrained as friction suddenly increased enormously in one case while the other case showed no abrupt changes. Most of the MMS-1 specimens showed irregular behavior only due to surface damage (elaborated in Section 3.2.4), and an indication of steady-state was observed after 20 to 30 μm of sliding displacement. Such behavior is comparable with natural completely decomposed volcanic granules [75], silt-coated quartz sand grains [85] and pumice [66]. Nevertheless, characteristics such as the friction values at the steady-state sliding, the non-linearity of the hardening regime and the type of surface damage are significantly different for the MMS-1 grains. One particular case of MMS-1 specimens had very different behavior, showing significantly higher tangential load compared to the other samples but without macroscopic damage of the grains. This behavior was attributed to the peculiar grain structure (of one of the two grains of the specimen) without the presence of any micro- or macro-vesicles and coating on the surface of the grain (inset of Figure 6a), resulting in significantly higher tangential loads, and the undulations in the load-displacement curves can be attributed, predominantly, to the morphology of the grains.

Figure 6b shows the mobilized friction coefficient (μ_mob_) values for MMS-1 grains; the steady-state friction coefficient values ranged between 0.55 and 0.80, excluding the cases which had macroscopic fragmentation and the case with hard MMS-1 grain. These two cases showed values of the coefficient of friction as high as 1.1 and as low as 0.3, respectively. The most common range of interparticle friction angles for MMS-1 grains tested in this study is 28.8–38.6°, where the range reported by Perko et al. [26] for friction angles of various regolith simulants was 33.7–53.3°, based on direct shear tests. Previous studies on Mars simulants have reported, based on macroscopic experiments, friction angles of around 35° [25,89], while Sullivan et al. [90], reported friction angles in a range of 30–37° based on wheel trenching sequences for Martian regoliths. It is noted that even though the bulk strength of granular materials, expressed with the macroscopic friction angle, is quantitatively related with the interparticle friction angle as multi-scale studies would suggest [30], the macroscopic friction results from the contribution of different mechanisms, including the interparticle friction, the resistance of the granular assembly because of particle interlocking as well as bulk dilation of the material [91].

Using similar interparticle loading experimental setups, Sandeep et al. [59] reported an average value of interparticle friction coefficient corresponding to the peak tangential load equal to 0.36 ± 0.09 (19.8°) for DNA-1A simulant, while He and Senetakis [66] reported an average friction angle of 38.3° for highly vesicular pumice. Thus, the results from the present study are, in general, closer to the reported friction angles for pumice, which material has similar textural characteristics with MMS-1 simulant (despite some fundamental differences as, for example, the mafic-based magmatic composition of MMS-1, whereas pumice is composed of felsic-based magma). MMS-1, pumice, and DNA-1A can be categorized as irregular-shaped grains with high surface roughness and interparticle friction angles. However, the primary differences in the Young’s moduli of these materials lead to varied frictional responses (as also suggested by [67]) in terms of load-displacement and stiffness-displacement curves. Much smaller friction angles (of the order of 5–17°) and regular trends in load-displacement curves have been reported for quartz sand grains, which materials have in general much smoother surfaces and much higher Young’s modulus [59,65,67,76].

#### 3.2.3. Normal versus Shearing Displacement

Delage et al. [25] reported significant compressive behavior of Martian simulants, and this phenomenon was also linked to the destabilizing and significantly higher friction values. In direct shear tests, the normal displacement variation can be attributed to the changes in the packing density of the grains (typically in terms of void ratio). In addition, the interparticle shearing tests on pumice grains by He and Senetakis [66] described the normal displacement variations during shearing and the effect of shearing cycles on the dilation tendency at the grain-scale. It was reported in [66] that asperity breakage in the initial shearing cycle initiated a compressive behavior, but the consecutive shearing cycles led to dilation and hence higher mobilization of tangential loads (and thus, higher mobilized interparticle friction). The general dilation or compression behavior in element tests (e.g., direct shearing) highly depends on the density of the sample and the normal stress applied. For example, a loosely packed dry sand sample in a shear box under lower normal stresses tends to compress during initial shearing, and once a denser packing is attained (decrease in void ratio), the sample dilates as the grains roll over each other. However, in a grain-scale test where a single grain pair is in peak-to-peak contact, the normal displacements (dilation or compression) are highly dependent on the micro-scale morphology of the grains. The surface asperities and their strength define whether the grains move away from each other (dilation) or towards each other (compression) to maintain the required constant normal load during shearing. In the present micromechanical tests on MMS-1 grains, the normal displacement variation was a result of asperity breakage and pore collapse on the grain surface, and significant compression was observed throughout the shearing process, which could explain, partly, the macroscopically compressive behavior reported by [24] on Mars simulant. Most cases in the present study showed partial dilation tendency either in the initial or later stages of shearing, but this behavior can be attributed to the morphology of MMS-1 grains, given their highly angular and irregular shapes. Another possible mechanism leading to partial dilation in the MMS-1 grains (particularly in the later stages of shearing) can be related to the saturation of pore collapse tendency or asperity breakage after initial sliding; these mechanisms alter the morphology of the particles leading to dilation tendency in subsequent cycles of shearing, additionally showing the highly complex behavior of the Mars regolith simulants.

**Figure 7:**
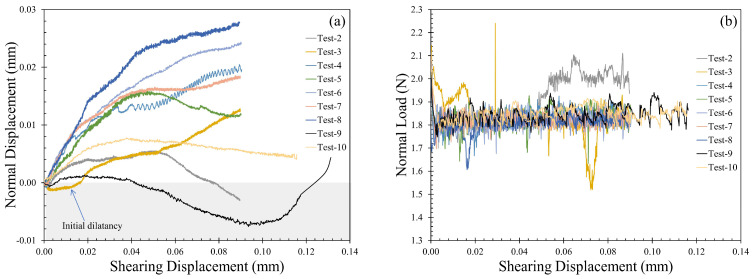
Variation of (**a**) normal displacement and (**b**) normal load against shearing displacement during sliding.

Figure 7a shows representative plots of the variation of normal displacement against shearing displacement for MMS-1 specimens. Only one test case showed initial dilation tendency (highlighted in blue in Figure 7a), but most cases showed initial compression, and later the normal displacements showed either saturation or reversal of the trend. These variations in the normal displacement result, solely from the damage of the MMS-1 grains during shearing and can be ascertained from the regularity in the normal load variation during shearing. Figure 7b shows the variation of normal load against shearing displacement for the MMS-1 grains tested, and most cases showed no abnormalities in the normal load. As the shearing tests were conducted under a constant normal load which was implemented in a force-controlled mode, the linear actuator guides the motion of the MMS-1 grain in the vertical direction to maintain the target normal load (around 2 N). However, some curves fluctuated during sliding, which is related with the influence of the morphological irregularities of some grains (as well as the weak nature of the particles) that led to either breakage of the specimen or a dilation tendency in some of the experiments (as shown in Figure 7a).

#### 3.2.4. Damage Analysis

**Figure 8:**
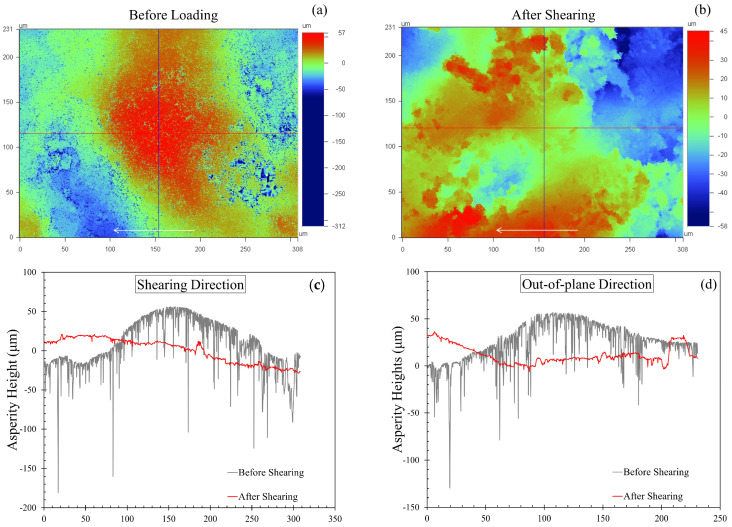
Surface profiles of a representative MMS-1 grain (**a**) before loading and (**b**) after shearing (arrows indicate shearing direction). The corresponding 2D profiles along (**c**) the shearing and (**d**) the out-of-plane direction.

In Section 3.2.2, different modes of damage and the corresponding variations in the tangential load-displacement curves were discussed. Only a few cases of the specimens subjected to shearing showed catastrophic failure, defined as fragmentation, which resulted in peculiar variations in the tangential load and hence in the mobilized friction—displacement curves. The majority of the cases showed surface damage in terms of asperity breakage and pore collapse, which led to irregularities in the load-displacement curves. Figure 8a,b shows the surface profiles of a representative MMS-1 grain before and after shearing, without any corrections for tilt and curvature. The profile in Figure 8a (before shearing) shows a peak on the surface in the vicinity of the contact region. In Figure 8b, the peak formation was eliminated completely due to the sliding, and this explains the macroscopic asperity breakage leading to a change of particle shape (locally) in the shearing region. The red and blue crosshairs in Figure 8a,b represent the locations where the two-dimensional profiles of the surface were captured both in the shearing and the out-of-plane directions. These 2D profiles before and after shearing are compared in Figure 8c,d.

A clear indication of peak removal was observed with a drop in the average height of around 25 μm, which is within the range of normal compression presented in Figure 8a. Even though the present experiments refer to highly irregular particles which showed, in many cases, severe damage on their surfaces because of the application of normal loading and shearing, the surface profile analysis in Figure 8 is in qualitative agreement with previous studies examining the changes of the roughness characteristics of quartz sand grains [92] and crushed limestone surfaces [93] on that peak asperities are in general removed and the roughness, on average, decreases after the completion of shearing.

In the research of saltation in Mars atmosphere, it was reported that the collision of grains during aeolian transport creates dust as they disaggregate [94]. With the atmospheric conditions on Mars, higher energetic collisions are expected, leading to more damage of the grains [95]. The present micromechanical (grain-to-grain interface shearing) tests also resulted in surface damage and the production of debris. These contacts can be related to the oblique impacts of the grains, but the inertial forces are not involved in the present study, which is one of the governing factors in particle collisions at different impact velocities. In addition, the size of grains considered in this study is large to be related with aeolian transported materials, but the damage mechanisms, micro-structural features and shearing mechanisms can be related with the particle collisions of Mars regoliths (which was out of the scope of the present study).

### 3.3. Repeated Monotonic Loading

**Figure 9:**
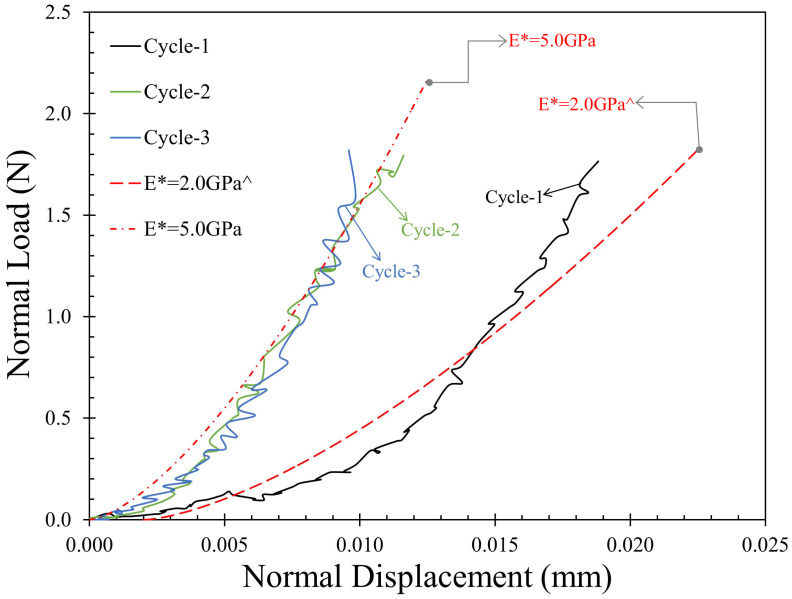
Variation of normal contact response with three cycles of repeated monotonic loading. (^ initial shift in normal displacement applied).

A representative set of repeated monotonic loading tests was conducted on MMS-1 grain pairs under a constant normal load of 2 N for three cycles. The cases which showed catastrophic damage of MMS-1 grains (as described in Section 3.2.2) were not selected for repeated loading. These repeated monotonic loading tests provide some additional insights on the contact response of granular materials under the combined influence of normal and tangential loading, taking into account the loading history [75,76]. Figure 9 shows the normal contact response of a representative case for three cycles of loading. The first cycle of normal loading is the “virgin loading” (without any previous loading history), while the second and third cycles have a history of one and two normal and tangential loading cycles, respectively. The second and third cycles of normal loading showed stiffer behavior compared to the first cycle, but no differences were observed between the second and third cycles, which implies that, perhaps, the most severe changes of the morphology of the grains took place in cycle-1 of loading (as a coupled influence of normal and tangential loading).

From the similar approximation of contact moduli with the Hertz model as shown in Figure 5a, the contact modulus was observed to increase from 2 GPa to 5 GPa beyond the first cycle. This increase can be attributed to the increase in contact stiffness from the damage of weak asperities in the first cycle of normal loading and shearing. It is well understood that the damage of asperities is more prominent in shearing than in normal loading as previous micromechanical-based studies would suggest [76], particularly at lower normal loads. Hence, the change in stiffness (reported as contact modulus) can be attributed, predominantly, to the applications of shearing in the first cycle. Similar observations were reported by Kasyap and Senetakis [77], where the shearing behavior of kaolinite-coated quartz sand particles was evaluated implementing a similar grain-scale methodology.

**Figure 10:**
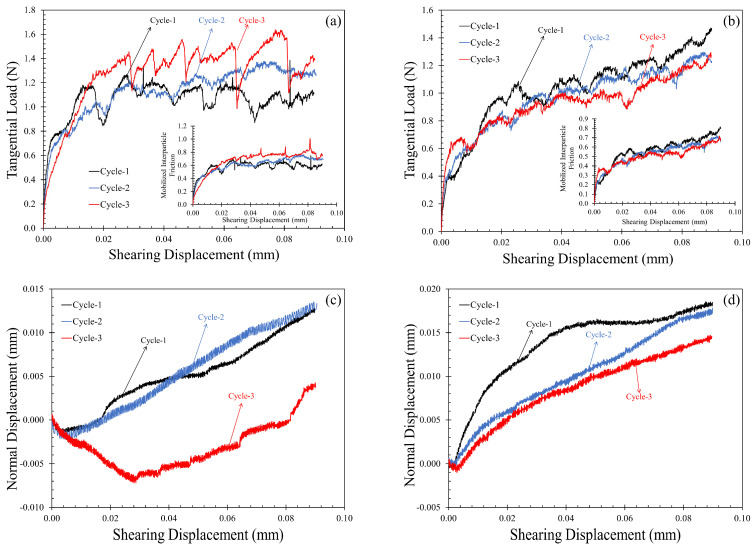
Tangential contact response of MMS-1 for three cycles of repeated monotonic loading for two cases of MMS-1 specimens showing opposite trends: (**a**,**b**) tangential load-displacement curves (insets: mobilized friction versus tangential displacement) and (**c**,**d**) normal displacement versus shearing displacement curves.

Opposite to the clear influence of loading history in the normal direction, the shearing behavior did not show much difference between the three cycles of repeated monotonic loading (Figure 10a,b), except for a small percentage of increase in the steady-state coefficient of friction (around 25% compared to the first cycle) in the third cycle of shearing (Figure 10a). However, this increase is insignificant when compared with the undulations in a given tangential load-displacement curve and the variabilities observed in different MMS-1 specimens. In another repetition of the test with a different pair of grains (Figure 10b), the trend was observed to be opposite, i.e., the mobilized tangential load (and interparticle friction) decreased with increasing number of shearing cycles. The mobilized interparticle friction versus tangential displacement curves corresponding to the two test cases are plotted as insets in Figure 10a,b. It can be stated that the repetition of normal and tangential loading on the same paths resulted in varied behavior due to the highly irregular surfaces and vesicular structure of the MMS-1 grains. Given the irregularity in the load-displacement curves and the variation with repeating loading, the trend of mobilized tangential loads for a given pair of grains can be understood or justified from the normal displacement variation during shearing. Figure 10c,d show the variation of normal displacement during shearing corresponding to the test cases discussed in Figure 10a,b, respectively, which showed opposite trends (dilation and compression) in the normal displacements.

In the dilation case (Figure 10c), as the number of repeated monotonic loading cycles increased, particularly the third cycle, the specimen showed significant dilation tendency (possibly due to grain morphology changes from the loading history), leading to higher mobilization of tangential loads. On the contrary, Figure 10d showed completely compressive behavior during shearing, and the amount of compression reduced with the number of loading repetitions. The third cycle of Figure 10d showed a slight indication of dilation in the initial stages of shearing, and the corresponding increase in tangential loads can be observed before 10 μm of sliding displacement in Figure 10a. These results suggest the potential strong relationship between frictional response and dilation/compression (or frictional response–dilation–loading history) at the grain-scale, which has been majorly overlooked in previous micromechanical studies.

## 4. Conclusions

The micromechanical behavior of MMS-1 (Mojave Mars Simulants) was investigated in the present study with emphasis on the frictional behavior of pairs of grains subjected to normal and tangential loading. Basic chemical characterization of MMS-1 was performed with EDS and the results from this analysis were compared with other simulants reported in the literature. Grain shape and surface characterizations revealed that MMS-1 grains are highly angular and irregular in terms of surface morphology with vesicles and protrusions on their surface. Single grain crushing strength tests on MMS-1 showed that the material has lower strength than completely decomposed granitic grains, and three variants of crushing modes were also identified. The interparticle normal loading tests showed a contact modulus range of the order of 1.5–5.0 GPa and increasing the contact load to 5N resulted in catastrophic damage of the grains. The interparticle friction angle values ranged between 27.5° and 41.0° (μ = 0.52–0.87) while the friction angles measured from direct shear tests in previous studies were reported as 33.3°–53.3° (μ = 0.66–1.34). Most of the tests showed microscopic damage of the surfaces, and surface profile observations showed that the peak of the grains was completely damaged after shearing. A few specimens showed fragmentation in the contact zone, leading to abnormal deviations in the friction values. The normal displacement variation during shearing displayed a completely compressive behavior owing to the pore collapse and asperity damage in the contact zone. A representative set of repeated monotonic loading tests showed increased stiffness in the normal direction. However, in the tangential direction, the influence of loading history did not follow a monotonic trend and the increase or decrease in the mobilized friction in repeated shearing cycles was related with the tendency of dilation or compression of the specimens. The results from the present study can provide enhanced modeling and direct input parameters in the discrete-based simulation of Mars surface, the analysis of rover wheel-soil interaction as well as modeling in problems related with transportation and geo-environmental processes on the surface of the Mars.

## Figures and Tables

**Table 1:** Element weight percentages of MMS-1 grains obtained from EDS analysis in comparison with other simulants.

Element	JSC Mars-1 ^#^	MMS *	MMS-1 ^	Present Study on MMS-1				
				1	2	3	4	5
Si	24.92	26.11	31.1	32.1 ± 1.2	32.6 ± 1.1	30.4 ± 1.5	28.5 ± 1.6	30.3 ± 1.4
O	46.08	45.68	43.3	31.0 ± 1.5	34.2 ± 1.4	31.3 ± 2.0	33.0 ± 2.3	31.8 ± 1.9
Fe	5.19	3.23	2.78	19.5 ± 1.9	16.6 ± 1.6	19.4 ± 2.5	17.8 ± 2.8	18.9 ± 2.4
Al	8.45	6.03	4.67	5.8 ± 0.5	5.7 ± 0.4	5.8 ± 0.6	7.3 ± 0.8	6.3 ± 0.6
Ca	5.68	9.05	4.36	4.5 ± 0.6	3.5 ± 0.5	4.7 ± 0.8	4.8 ± 0.9	4.7 ± 0.8
K	0.45	0.29	1.29	2.2 ± 0.4	2.5 ± 0.4	2.6 ± 0.6	2.9 ± 0.7	2.6 ± 0.6
Mg	4.65	6.18	4.28	2.1 ± 0.3	2.2 ± 0.3	1.9 ± 0.4	3.4 ± 0.5	2.5 ± 0.4
Na	2.06	2.67	3.51	0.9 ± 0.3	1.2 ± 0.3	1.5 ± 0.4	0.2 ± 0.4	0.9 ± 0.4
Ti	2.00	0.56	0.58	0.9 ± 0.5	0.4 ± 0.5	1.6 ± 0.8	1.8 ± 0.8	1.4 ± 0.7
Mn	0.21	0.13	0.08	0.5 ± 0.8	1.2 ± 0.8	0.0 ± 0.0	0.0 ± 0.0	0.2 ± 0.3
P	0.18	0.04	0.04	0.2 ± 0.2	0.0 ± 0.2	0.4 ± 0.3	0.2 ± 0.4	0.3 ± 0.3
Cr	0.01	0.02	-	0.1 ± 0.7	0.0 ± 0.0	0.1 ± 0.1	0.0 ± 0.0	0.1 ± 0.6
S	0.11	0.03	-	0.0 ± 0.2	0.0 ± 0.0	0.4 ± 0.3	0.0 ± 0.3	0.1 ± 0.3

* after Peters et al. [20]: ICP-AES method, ^#^ after Morris et al. [68]: XRF method, ^ after Caporale et al. [69].

**Table 2:** Testing details and preliminary observations.

S.No	F_N_ (N) ^#^	δ_N,max_^@^ (mm)	F_T,SS_ (N)	μ_SS_	φ (deg.)	δ_N,shear_ (μm) *	
						Dilation	Compression
1	2.06	0.065	1.47	0.70	35.0	0	24.5
2	1.90	0.014	2.28 ^&^	1.14 ^&^	47.7 ^&^	−3.2	5.5
3 ^	1.84	0.024	1.39	0.61	31.4	−1.4	13.4
	1.85	0.012	1.38	0.60	30.9	−2.3	13.5
	1.90	0.008	1.64	0.79	38.3	−7.2	4.3
4	1.83	0.084	1.26	0.70	35.0	−0.9	20.5
5	1.83	0.020	1.51	0.81	39.0	0	22.7
6	1.82	0.014	0.95	0.52	27.5	−0.1	24.4
7 ^	1.83	0.019	-	-	-	−0.5	24.9
	1.83	0.012	-	-	-	−0.3	17.8
	1.83	0.010	-	-	-	−1.1	14.7
8	1.81	0.026	0.62 ^&^	0.34 ^&^	18.8 ^&^	0	27.9
9	1.84	0.048	1.45	0.79	38.3	−7.6	4.4
10	1.86	0.026	1.59	0.87	41.0	−0.1	22.1
11	1.95	0.035	1.64	0.84	40.0	0	19.7
12	1.85	0.022	1.55	0.84	40.0	0	23.4
13	1.87	0.029	1.27	0.68	34.2	−0.1	21.9
14	1.91	0.041	1.44	0.75	36.9	−0.1	17.8

# average values of normal load during shearing, ^@^ maximum normal deformation during normal load application, ^ Repeated monotonic loading test cases, ^&^ significant fragmentation led to abnormal behavior, * normal displacement range during shearing.

## Data Availability

Data are available by the corresponding author after reasonable request.

## Abbreviations

MMS	Mojave mars simulant
SEM	Scanning electron microscope
EDS	Energy dispersive X-ray Spectroscopy
FSO	Full scale output
F_N_	Normal Load
δ_N_	Normal displacement
F_T_	Tangential Load
δ_T_	Tangential displacement
δ_N,max_	Normal displacement for a given normal load
δ_N,shear_	Normal displacement during shearing
μ_SS_	Steady state value of interparticle friction coefficient
φ	Interparticle friction angle.
μ_mob_	Mobilized interparticle friction
F_C_	Crushing load
σ_c_	Characteristic crushing strength
d	Geometric mean of smaller SCC dimensions
P_S_	Survival probability
σ_p_	Peak stress
E*	Contact modulus
m	Weibull modulus
S_q_	Surface roughness
